# Reply to: On the statistical foundation of a recent single molecule FRET benchmark

**DOI:** 10.1038/s41467-024-47734-2

**Published:** 2024-04-30

**Authors:** Markus Götz, Anders Barth, Søren S. -R. Bohr, Richard Börner, Jixin Chen, Thorben Cordes, Dorothy A. Erie, Christian Gebhardt, Mélodie C. A. S. Hadzic, George L. Hamilton, Nikos S. Hatzakis, Thorsten Hugel, Lydia Kisley, Don C. Lamb, Carlos de Lannoy, Chelsea Mahn, Dushani Dunukara, Dick de Ridder, Hugo Sanabria, Julia Schimpf, Claus A. M. Seidel, Roland K. O. Sigel, Magnus B. Sletfjerding, Johannes Thomsen, Leonie Vollmar, Simon Wanninger, Keith R. Weninger, Pengning Xu, Sonja Schmid

**Affiliations:** 1 PicoQuant GmbH, Rudower Chaussee 29, 12489 Berlin, Germany; 2 Department of Bionanoscience, Kavli Institute of Nanoscience Delft, Delft University of Technology, Van der Maasweg 9, 2629 HZ Delft, The Netherlands; 3 Department of Chemistry, University of Copenhagen, 2100 Copenhagen, Denmark; 4 Novo Nordisk Center for optimised oligo escape and control of disease University of Copenhagen, 2100 Novo Nordisk Foundation Centre for Protein Research, Faculty of Health and Medical Sciences, University of Copenhagen, 2100 Copenhagen, Denmark; 5 Department of Chemistry, University of Zurich, 8057 Zurich, Switzerland; 6 Laserinstitut Hochschule Mittweida, University of Applied Sciences Mittweida, 09648 Mittweida, Germany; 7 Department of Chemistry and Biochemistry, Ohio University, Athens, OH USA; 8 Physical and Synthetic Biology, Faculty of Biology, Ludwig-Maximilians-Universität München, Großhadernerstr. 2-4, 82152 Planegg-Martinsried, Germany; 9 Department of Chemistry, University of North Carolina, Chapel Hill, NC 27599 USA; 10 Lineberger Comprehensive Cancer Center, University of North Carolina, Chapel Hill, NC 27599 USA; 11 Department of Physics and Astronomy, Clemson University, Clemson, SC 29634 USA; 12 Department of Biochemistry and Molecular Pharmacology, New York University School of Medicine, New York, NY USA; 13 Institute of Physical Chemistry, University of Freiburg, Freiburg, Germany; 14 Signalling Research Centers BIOSS and CIBSS, University of Freiburg, Freiburg, Germany; 15 Department of Physics, Case Western Reserve University, Cleveland, OH USA; 16 Department of Chemistry, Case Western Reserve University, Cleveland, OH USA; 17 Department of Chemistry and Center for Nano Science (CeNS), Ludwig Maximilians-Universität München, Butenandtstraße 5-13, 81377 München, Germany; 18 Bioinformatics Group, Wageningen University, Droevendaalsesteeg 1, 6708PB Wageningen, The Netherlands; 19 Department of Physics, North Carolina State University, Raleigh, NC 27695 USA; 20 Spemann Graduate School of Biology and Medicine (SGBM), University of Freiburg, Freiburg, Germany; 21 Institut für Physikalische Chemie, Lehrstuhl für Molekulare Physikalische Chemie, Heinrich-Heine-Universität, Universitätsstr. 1, 40225 Düsseldorf, Germany; 22 NanoDynamicsLab, Laboratory of Biophysics, Wageningen University, Stippeneng 4, 6708WE Wageningen, The Netherlands; 23 Present Address: Department of Chemistry, University of Basel, Basel, Switzerland

**Keywords:** Fluorescence resonance energy transfer, Single-molecule biophysics

**replying to** A. Saurabh et al. *Nature Communications* 10.1038/s41467-024-47733-3 (2024)

In their ‘Matters Arising’ manuscript, Saurabh et al. discuss two issues related to single-molecule Förster resonance energy transfer (smFRET) experiments: the use of the Gaussian noise approximation and spectral crosstalk. Their arguments are based on simulations obtained with parameters that differ significantly from the typical conditions measured experimentally, and, thus, from the regime included in the original study (Götz et al.^1^). In addition, they make claims about our multi-lab blind study that we would like to rectify. In Table 1, we provide a list of specific statements made by Saurabh et al. with our respective explanations.

**Table 1:** Specific statements by Saurabh et al. with respective explanations

#	Quote:	Reply:
**1)**	*“These sources include photon shot noise, detector noise, and spectral crosstalk (…) the data generated by the competition organizers lacked these features.”*	Our study does incorporate all key features that are relevant for the vast majority of experimental smFRET literature. As detailed in our reply, in this regime: (a) shot noise is included with a mathematically justified Gaussian noise model; (b) detector noise just broadens the Gaussian distribution and, thus, is also included; and (c) crosstalk is irrelevant since kinetic rates are investigated (not FRET-derived distances).
**2)**	“*the benchmark is setup in such a way that* […] *tools incorporating physical features beyond the Gaussian noise model result in inaccuracies*”.	The simulations necessary to justify such a statement are missing from Saurabh et al. This would require a comparison of simulations with both a Gaussian and a Poissonian noise model at similar low count rates. In fact, Fig. 1a–d and Fig. 2a–d demonstrate an equivalent performance of both HMM variants (with Gaussian and Poisson emission HMMs) at count rates >50 photons per bin. Instead, Saurabh et al. unintentionally demonstrate that the benchmark design allowed for a fair comparison of tools with different noise models.
**3)**	*” the benchmark participants exhibit important biases in the presence of spectral crosstalk, one of many widespread FRET features unaccounted for in the competition”*	This is an unsubstantiated comment. Spectral crosstalk is not relevant for the kinetic analysis, as visible in Fig. 2c, d by Saurabh et al. In addition, simply stating “*one of many widespread FRET features*” is ambiguous, and we cannot scientifically reply to this.
**4)**	“*For EMCCD cameras… Convolving these distributions leads to a final noise model often inadequately approximated by a Gaussian*.”	This statement requires justification. It fully conflicts with existing literature^18,19^ investigating the actual *experimental* camera noise at the low and high signal-to-noise regime.
**5)**	*“*Figure 1 *also highlights the failure of the Gaussian emission HMM in analyzing data generated with Poisson emission:”*	This statement does not follow from their simulation. The problem of the HMM likely arises due to the unrealistically poor signal-to-noise ratio and not the noise model. To substantiate their statement, the authors would need to run a simulation with Gaussian noise at the same noise level, but this data is missing and hence the comparison is unsystematic and meaningless.
**6)**	*“Spectral crosstalk is another critical example of a physical feature often incorporated in quantitative FRET analyses [10] and corrects for photon misidentification. Ignoring crosstalk in analysis tools leads to FRET efficiency estimate biases confounding quantitative FRET pair distance assessments [11]. “*	These statements are irrelevant to the analysis of the kinetic information within the trace.
**7)**	*“However these photons are not correctly added back as part of donor intensity.”*	This statement is truly surprising because it ignores the major developments in the field of the past decades^20–24^. Crosstalk correction is now well understood and standardized^7,8^, and this statement about ‘adding photons back’ conflicts with all established procedures. The gamma factor simultaneously corrects measured FRET efficiency for quantum yields as well as the limited spectral pass-band of both donor and acceptor channel filters. It corrects for all the donor photons that fall outside the donor pass-band, which is a complete correction^25^. Plus, by scaling the donor photons, one changes the noise characteristics of the data, which can falsify further downstream analyses.
**8)**	*Figure 2*.	We are convinced that the large discrepancy in FRET efficiency values displayed in Figure 2e,f is incorrect but, since specifics on the methods used by Saurabh et al. are lacking, we and others cannot verify the correct application of the detection correction factors.

In our reply, we will discuss three points, summarized here and detailed below.

1. smFRET trajectories from typical surface-tethered experiments are well described by Gaussian noise models (mean photon counts are >50 per data point). Non-Gaussian Poisson noise only becomes relevant for smFRET data with extremely low photon counts, which is generally avoided by increasing the laser power and/or integration time of the experiment.
2. Spectral crosstalk correction is relevant for determining correct FRET efficiencies and FRET-derived distances, but it does not impact the kinetic rate derivation, which is the focus of Götz et al.
3. The study of Götz et al. compares the strengths and weaknesses of currently available kinetic tools to draw lessons for further development. It does not “*favor*” any approaches or “*lead to bias*” etc. as incorrectly stated by Saurabh et al. Saurabh et al. are welcome to conduct dedicated studies on the specific features they propose to extend the work of Götz et al.

**1) Single-molecule FRET trajectories from typical surface-tethered experiments are well described by Gaussian noise**. While photon-counting detection is a Poissonian process, the photon count rates in these experiments are sufficiently large for the Gaussian approximation to be valid: commonly used smFRET trajectories show mean photon counts of >50 or even several hundred photons per data point. In the words of Taekjip Ha’s seminal paper ‘*A practical guide to single-molecule FRET’:* “*To achieve adequate signal-to-noise ratio, ~100 total photons need to be detected*.”^2^. This is the most common experimental regime and is considered in Götz et al. Specific literature examples report 100–500 photons^3^ and 100–300 photons^4^ for camera-based experiments and for APD-based experiments 100-400 photons^5^. These count rates are perfectly in line with “*the limit of longer camera exposures*” mentioned by Saurabh et al., where the “*Gaussian approximation [is] warranted*” even when a Poisson process, such as shot noise, is involved. For illustration, Fig. 1 shows an overlay of Poissonian and Gaussian probability density functions and their agreement for the case considered here and in our published work^1^: >50 photons per time bin.

**Fig. 1:**
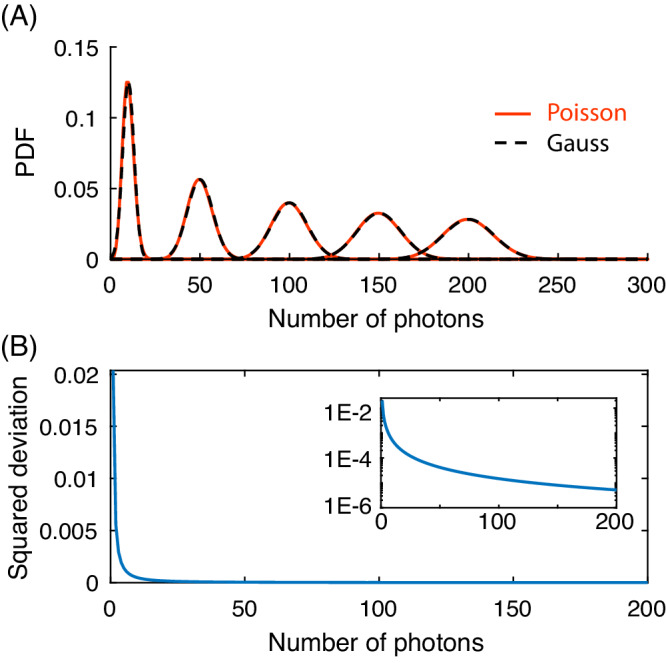
Comparison of Poissonian and Gaussian noise models as a function of photon counts. **A** Overlay of Poissonian and Gaussian probability density functions (PDF) for mean photon counts of 10, 50, 100, 150, and 200. Differences between the PDF pairs are hardly visible. **B** The squared deviation of the Poissonian versus Gaussian PDF is displayed for mean photon counts of 1–200. The inset shows the same plot in logscale.

In light of this, the Gaussian simulation by Saurabh et al. (mean photon counts of 58 to 152) reflects the typical experimental smFRET trajectories well, and both analysis routines based on Gaussian and Poissonian noise work equally well in this case. Moreover, Saurabh et al. demonstrate that for a Poissonian noise simulation (Fig. 2) with comparable mean photon counts of 58–141, both analysis approaches (Gaussian and Poissonian) extract equally accurate rates. Saurabh et al. state accordingly: “*All methods considered (…) predicted equivalently good state trajectories*” (Fig. 2 caption), which again demonstrates the validity of the Gaussian approximation in this regime. It also validates the benchmark design, demonstrating that it allowed for a fair comparison of tools employing different noise models. Moreover, Saurabh et al. directly disprove their own claim that “*the benchmark is setup in such a way that* […] *tools incorporating physical features beyond the Gaussian noise model result in inaccuracies*”. In fact, even for low intensities Saurabh et al.’s claim stays unsupported by any data, since they don’t compare simulations using Gaussian and Poissonian noise for low intensities.

Beyond the Gaussian regime, Saurabh et al. choose to simulate data in the extreme low-signal regime of 5–12 photons, showing the expected non-Gaussian Poisson noise. This regime is interesting in specialized cases such as single-photon-counting experiments with sub-millisecond dynamics, but that is not the topic of the Götz et al. study. Instead, Götz et al. consider the most common case of smFRET studies with surface-tethered molecules and time-binned trajectories. To avoid low signal in such experiments, one would simply increase the laser power or integration time to achieve better quality data than the one simulated by Saurabh et al. in Fig. 1g. It is evident from Fig. 1g that the experimental trajectories with such low photon counts exhibit very poor signal-to-noise ratio. Not only are the analysis of such traces prone to experimental artifacts, there is also a high probability that such traces are not coming from the molecule of interest but rather, for example, dirt. Therefore, in experimental works, such poor time-binned data are not credible and hence not used by the community. Importantly, time-binned data should not be confused with more specialized single-photon-counting experiments that are analyzed using photon-by-photon analyses^6^, which is an entirely different method not covered in our Götz et al. study. We note further that Götz et al. specifically discuss the breakdown of the Gaussian approximation in the extreme low-signal regime: “… *at 1* *kHz sampling [instead of 0.1* *kHz], the data shows single-photon discretization and non-Gaussian noise (Supplementary Fig. 2a, b), thus deviating from the basic assumptions underlying most of the considered analysis tools. Indeed, the overall agreement of the rate constants at this lower SNR was reduced*”. While this extreme low-signal regime is of niche relevance, our multi-lab blind study—as a foundation for future benchmarks—focuses on the most common scenarios found in experimental smFRET studies. We deliberately avoided extreme conditions that some tools could specialize in and others could not. This choice will certainly not limit any tool to demonstrate and pursue extreme applications in other projects.

In summary, we clearly show here that there is no controversy: (i) the choice of the simulated (Gaussian) noise regime by Götz et al. reflects the majority of cases found in literature; (ii) it is perfectly valid that some analysis tools (not all) make use of this general knowledge about smFRET noise characteristics, (iii) the issue raised by Saurabh et al. stems from their special choice of simulation parameters, and (iv) the benchmark allowed for a fair comparison as supported by Saurabh et al.’s Fig. 2c, d. Furthermore, Götz et al. also include several experimental datasets—not just simulations. (All data are publicly available).

**2) Spectral crosstalk corrections** are relevant for accurate FRET efficiency determination and FRET-derived distances, as benchmarked by many of us elsewhere^7,8^, but they are irrelevant for the analysis of kinetic rate constants. In fact, kinetic analyses are often intentionally applied to uncorrected intensity traces to retain the original noise characteristics. Determining accurate FRET efficiencies and kinetic rate constants are two independent tasks, often performed using separate software tools: e.g., iSMS^9^, TwoTone^10^, PAM^11^, etc. for accurate FRET, whereas HaMMy^12^, STaSI^13^, SMART^14^, ebFRET^15^, etc. for kinetics. Götz et al. discuss the latter task: kinetics. So, again, we cannot identify any controversy since Saurabh et al. also nicely demonstrate that kinetic rate constants are not affected by spectral crosstalk corrections, as shown by the gray vs. blue data in Fig. 2c, d.

Regarding the specific comments on Hidden Markury and MASH-FRET, we note that the Hidden Markury package does not include FRET efficiency corrections. Hence, it is not meaningful to assess Hidden Markury’s ability to do such corrections as done by Saurabh et al. in Fig. 2e, f. Similarly, Saurabh et al. makes strong claims concerning MASH-FRET, where Saurabh et al. claimed to identify problems (Fig. 2e, f), which, however, cannot be verified since Saurabh et al. does not provide their code at the moment of this Reply. Notably, MASH-FRET had been described and assessed in all detail in a previous peer-reviewed publication^16^, which Saurabh et al. disregard. In addition, we now re-assessed MASH-FRET using the experimental data of the well-established ‘FRET standard’ publication^7^ (publicly available). In contrast to the unverifiable claim in Fig. 2e, f by Saurabh et al., we find that MASH-FRET yields accurate FRET efficiencies of 0.15 ± 0.01 (published: 0.15 ± 0.02^7^) and 0.54 ± 0.03 (published: 0.56 ± 0.03^7^). Method details: donor leakage = 0.07, acceptor direct excitation = 0.065, gamma = 1.14, mean values and standard deviations determined using bootstrapping of 59–107 FRET trajectories following ref. ^17^. In summary, the MASH-FRET procedure follows the established corrections for accurate FRET^7^, and the software provides accurate FRET efficiencies when used correctly.

In addition, we note that the suggestion to add crosstalk photons back to the donor channel is, at best, useless, if not worrisome, as it destroys the photon statistics (useful for further analyses). The impact of such crosstalk on the FRET efficiency is commonly corrected using a set of correction factors (such as the ‘gamma factor’ as repeatedly described previously, incl. in ref. ^7^), but, importantly, without “*adding back*”—in reality, rescaling—the donor photons as suggested by Saurabh et al. Either way, the correction of crosstalk is not relevant to the conclusions of the Götz et al. paper.

**3) The Götz et al. study compares the strengths and weaknesses of currently available kinetic tools**, to draw lessons for further development. It does not “*favor*” any approaches or “*lead to bias*” etc., as nicely verified by Saurabh et al. in their Fig. 1a–f and Fig. 2c, d. The Götz et al. study was conducted over the course of three years in a fully transparent way, including published raw data, code, simulation parameters, and peer review files. In contrast, a complete description of the simulations and analysis by Saurabh et al. is missing at the time of this Reply, preventing an independent replication and verification of their results. Furthermore, Götz et al. clearly describe the participant’s prior knowledge in each round of the blind study, whereas many published benchmarks are not conducted as blind studies. Ultimately, based on the comparison of three simulated and four experimental datasets evaluated in Götz et al., forward-looking suggestions could be made to accelerate the scientific progress in the field.

Altogether, we thank the authors of Saurabh et al. for their remarks and thoughts, which testify to the importance of the Götz et al. paper, and we look forward to future studies that build upon our and other works to jointly move the smFRET field forward. We were happy to see that the future directions identified by Götz et al.—uncertainty estimation, model selection, and state determination—were reiterated in the last paragraph by Saurabh et al., showing good agreement about the most relevant future developments.

## Data Availability

No new data were generated for this reply. All data are publicly available at the original publication: 10.1038/s41467-022-33023-3.
